# Noninvasive sampling reveals population genetic structure in the Royle’s pika, *Ochotona roylei*, in the western Himalaya

**DOI:** 10.1002/ece3.4707

**Published:** 2018-12-26

**Authors:** Sabuj Bhattacharyya, Farah Ishtiaq

**Affiliations:** ^1^ Centre for Ecological Sciences Indian Institute of Science Bangalore India

**Keywords:** Alpine, bottleneck, India, *Ochotona roylei*, population genetics, Royle’s pika, western Himalaya

## Abstract

Understanding population genetic structure of climate‐sensitive herbivore species is important as it provides useful insights on how shifts in environmental conditions can alter their distribution and abundance. Herbivore responses to the environment can have a strong indirect cascading effect on community structure. This is particularly important for Royle's pika (Lagomorpha: *Ochotona roylei*), a herbivorous talus‐dwelling species in alpine ecosystem, which forms a major prey base for many carnivores in the Himalayan arc. In this study, we used seven polymorphic microsatellite loci to detect evidence for recent changes in genetic diversity and population structure in Royle's pika across five locations sampled between 8 and 160 km apart in the western Himalaya. Using four clustering approaches, we found the presence of significant contemporary genetic structure in Royle's pika populations. The detected genetic structure could be primarily attributed to the landscape features in alpine habitat (e.g., wide lowland valleys, rivers) that may act as semipermeable barriers to gene flow and distribution of food plants, which are key determinants in spatial distribution of herbivores. Pika showed low inbreeding coefficients (*F*_IS_) and a high level of pairwise relatedness for individuals within 1 km suggesting low dispersal abilities of talus‐dwelling pikas. We have found evidence of a recent population bottleneck, possibly due to effects of environmental disturbances (e.g., snow melting patterns or thermal stress). Our results reveal significant evidence of isolation by distance in genetic differentiation (*F*_ST_ range = 0.04–0.19). This is the first population genetics study on Royle's pika, which helps to address evolutionary consequences of climate change which are expected to significantly affect the distribution and population dynamics in this talus‐dwelling species.

## 1 INTRODUCTION

Over the past century, climate has been changing at a faster pace in alpine habitats than in other ecosystems, for which many species must either adapt or migrate to areas with optimal conditions (Naftz et al., 2002; Shrestha, Gautam, & Bawa, 2012). Among mountain‐dwelling species, pikas (*Ochotona* species), in particular, have experienced a significant contraction in their lower elevation range (Moritz et al. 2008). For example, climate‐induced heat (Beever, Brussard, & Berger, 2003), cold, or nutritional stress (Wilkening, Ray, Beever, & Brussard, 2011) led to historical extinctions of local populations and recent range contraction. While dispersal to higher elevation may allow pikas to persist in suitable microclimate, habitat fragmentation or non‐availability of preferred food plants can impair their ability to cope with the changing environment and pose a threat to their survival and fitness (Bhattacharyya, Dawson, Hipperson, & Ishtiaq, 2018; Ray, Beever, & Loarie, 2012; Schloss, Nuñez, & Lawler, 2012; Walther et al., 2002).

The Royle's pika (*Ochotona roylei*) is a widespread small herbivore, occupying much of the Himalayan arc—from northwestern Pakistan to India (Jammu and Kashmir, Himachal Pradesh, and Uttarakhand), Nepal, and adjacent Tibet (Bhattacharyya & Smith, 2018) and inhabits rocky boulder or talus habitat between 2,400 and 5,200 m elevation. It is an asocial mammal (Bhattacharyya & Smith, 2018, Figure 1) and, unlike other talus‐dwelling pika species (e.g., American pika *Ochotona princeps*, Collared pika *Ochotona collaris*), does not exhibit haying activity to store winter food resources (Bhattacharyya, Adhikari, & Rawat, 2013). The Royle's pika usually occurs at low population densities from 12.5 per ha in Nepal to 16.2 per ha in the western Himalaya in India (Bhattacharyya, Adhikari, & Rawat, 2014a). Furthermore, the interannual variation in population density of the Royle's pika is often influenced by snow melting pattern, food availability, and refuge from predation risk (rock cover) (Bhattacharyya et al., 2014a). The Royle's pika is sensitive to high temperatures (>15°C) that lead to thermal stress and limit overall activity (Bhattacharyya, Adhikari, & Rawat, 2014b). Furthermore, the Royle's pika play a crucial role in trophic interactions in alpine habitat—pikas are the major prey base for many carnivores as well as influencing native plant community structure (Aryal, Sathyakumar, & Kreigenhofer, 2010; Bhattacharyya et al., 2013, 2018 ; Bhattacharyya, Dutta, Adhikari, & Rawat, 2015)—their disappearance can have a significant negative cascading effect on overall ecosystem functioning (Gilg, Sittler, & Hanski, 2009; Tylianakis, Didham, Bascompte, & Wardle, 2008).

**Figure 1:**
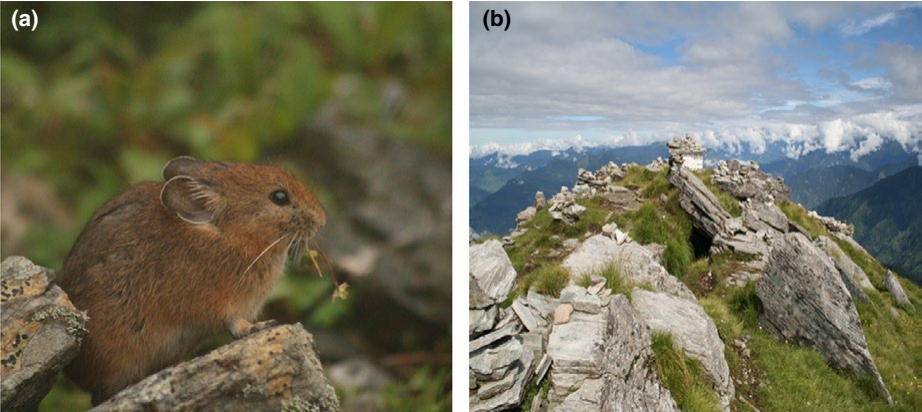
Royle's pika (a) and its talus habitat (b) in Kedarnath Wildlife Sanctuary, India. Photograph credit: Sabuj Bhattacharyya

The population genetic structure of small mammals is often influenced by landscape features and habitat fragmentation (Gerlach & Musolf, 2000; Peacock & Smith, 1997a; Peakall, Ruibal, & Lindenmayer, 2003). For talus‐dwelling species, topographical features (e.g., aspect) and alpine habitat connectivity play a crucial role in determining increased gene flow (e.g., American pikas; Castillo, Epps, Davis, & Cushman, 2014). Similarly, microhabitat characteristics such as rock cover area, availability of rock talus with small crevice size (<15 cm), govern the habitat occupancy in Royle's pika (Bhattacharyya et al., 2015). A large area of talus supports greater plant diversity and results in higher species richness in the pika's diet (Bhattacharyya et al., 2018). Therefore, we expect that Royle's pika populations would be influenced by topographical barriers (e.g., river low land valleys) as well as forage availability (e.g., C_3_ plants).

Philopatric settlements (dispersal very close to their natal territory) in pikas often lead to incestuous matings and low intrapopulation genetic variability (Henry, Sim, & Russello, 2012; Peacock & Smith, 1997a, 1997b ; Smith & Weston, 1990). While we lack information on sex‐biased dispersal or philopatry in Royle's pika, being a rocky talus obligate species, Royle's pika could have high inbreeding coefficients and relatedness across spatially close talus habitats or signatures of historical connectivity in habitat.

Population dynamics of Royle's pika are often influenced by snow melting pattern as snow acts as a thermal insulator to pika and their food plants (Bhattacharyya et al., 2014a). With recent (1982–2006) changes in snow patterns and rising temperatures in winters in the Himalaya region (Shrestha et al., 2012), these could result in patchy distribution of pika, loss of genetic diversity, and a demographic bottleneck in the species (Bhattacharyya et al., 2014a).

In the Himalayan arc, seven *Ochotona* species have been reported (Smith, Formozov, Hoffmann, Changlin, & Erbajeva, 1990); however, detailed long‐term studies on pika ecology (see Bhattacharyya et al., 2013; Bhattacharyya et al., 2014a; Bhattacharyya et al., 2014b; Bhattacharyya et al., 2015) and population genetics are yet to be considered. A fine‐scale pattern of distribution and habitat selection of the Royle's pika has been studied in the western Himalaya for the past nine years (Bhattacharyya et al., 2013, 2014a, 2014b, 2015 ). Using genetic markers in combination with fine‐scale spatial ecological data provides a unique opportunity to explore the genetic structure, patterns of relatedness, and demographic responses to changing environment. We employed a panel of seven polymorphic markers (see Table 1 for details) to understand the population structure and gene flow in the Royle's pika in the western Himalaya. We estimated genetic diversity between and within populations and explored evidence of inbreeding, isolation by distance, population genetic bottleneck, all of which will provide insights on how shifting environmental conditions can influence species distribution, overall fitness, and adaptive evolutionary potential in the face of climate change.

**Table 1:** Genetic diversity in Royle's pika populations in the western Himalaya

Loci^a^	A	AL	*H*_O_	*H*_E_	PIC	NF	*T*_m_	Reference
Ocp6	5	198–212	0.72	0.75	0.71	0.01	58	Peacock et al., 2002
Ocp16	4	113–127	0.43	0.48	0.4	0.04	58	Castillo et al., 2014
P7	8	144–176	0.68	0.84	0.82	0.09	62	Li et al., 2009
STR14	11	112–182	0.64	0.86	0.85	0.14	TD	Alves et al., 2015
SAT4	5	142–222	0.7	0.78	0.75	0.05	TD	Mougel et al., 1997
SAT3	6	120–186	0.7	0.77	0.74	0.05	TD	Mougel et al., 1997
STR31*	6	124–184	0.7	0.79	0.79	0.05	TD	Alves et al., 2015

Notes

A: mean number of alleles; AL: allele range in base pairs; *H*_E_: expected heterozygosity; *H*_O_: observed heterozygosity; NF: null allele frequency; PIC: polymorphism information content; TD: touchdown PCR; *T*_m_: annealing temperature in PCR.

a A panel of 64 microsatellite loci was selected for initial genotyping from following studies; American pika (*Ochotona princeps*,* n* = 25; Castillo et al., 2014; Peacock et al., 2002), Plateau pika (*Ochotona curzoniae, n* = 6; Li et al., 2009), Collared pika (*Ochotona collaris, n* = 5; Zgurski et al., 2009) as well as other lagomorph species such as European rabbit (*Oryctolagus cuniculus, n* = 28; Alves et al., 2015; Mougel et al., 1997) and a final set of seven polymorphic loci were selected based on amplification success rate across all populations.

^*^Significant deviation from HW equilibrium, *p* < 0.05.

## 2 MATERIALS AND METHODS

### 2.1 Field sampling

**Figure 2:**
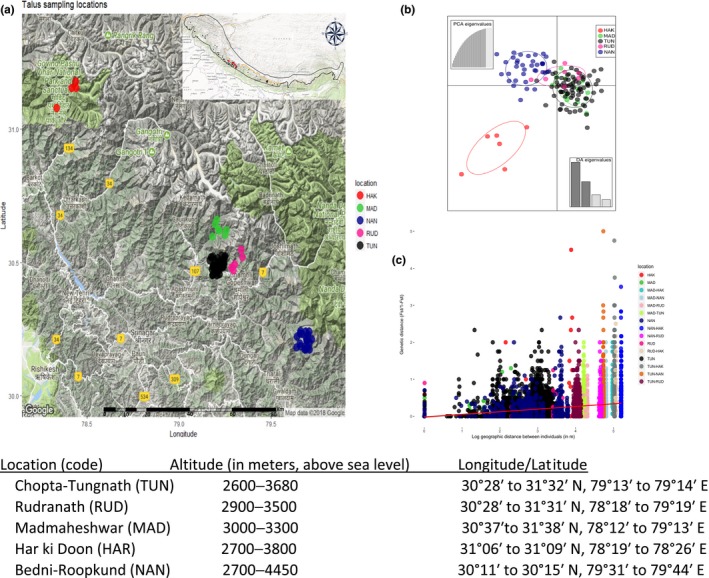
(a) Location of Royle's pika talus sampled for fecal pellets in Garhwal, Uttarakhand, India. Inset map showing distribution range of Royle's pika across Himalayan arc, black dots represent presence locations, red triangles represent sampled locations. (b) The scatterplot showing first two components of the discriminant analysis of principal component (DAPC) using pika locations (*K* = 5) as prior clusters. Each pika sampling location in DAPC was indicated by colored inertia ellipses and dots represent individuals. The first principal component PC I (abscissa) explains 50.21%, whereas the second principal component PC II (ordinate) explains 28.35% of the total genetic variance. (c) Isolation‐by‐distance analysis among individuals across sampled locations. Each color represents comparison within and across locations

The Royle's pika is usually found in talus habitats around forest trails with a home range of approximately 50 m^2^ (Bhattacharyya et al., 2015; Kawamichi, 1968). We surveyed talus habitats using noninvasive fecal sampling across five locations spanning an elevational gradient (2,600–4,450 m above sea level): Kedarnath Wildlife Sanctuary (Chopta–Tungnath [TUN], *n* = 105; Rudranath [RUD], *n* = 10; Madmaheshwar [MAD], *n* = 17), Govind Wildlife Sanctuary (Har ki Doon [HAR] *n* = 22), Nanda Devi Biosphere Reserve (Bedni‐Roopkund [NAN], *n* = 69) in Garhwal region, Uttarakhand, India (Figure 2a).

Fecal pellets were collected in two seasons: postmonsoon (late October–mid‐November 2014), and premonsoon (late May–June 2015). The talus size varies between 10 and 200 m^2^ and a 50 m^2^ plot was laid in each talus, which was thoroughly searched for fecal pellets. In order to maximize our chances of sampling different individuals, samples were collected from fecal piles at 50 m distance within a talus. Fresh fecal pellets (moist, dark brown/black in color) were collected in airtight plastic tubes with silica gel and were labeled with name of sampling location and geographic coordinates (latitude and longitude). We analyzed spatial data using the R package “geosphere” (Hijmans, Williams, & Vennes, 2015) and estimated distance between talus and sample locations (Figure 2a).

### 2.2 Molecular methods

The Royle's pika often feeds on plants with high levels of secondary metabolites (Bhattacharyya et al., 2013, 2018 ). The presence of plant secondary metabolites in fecal pellets can hamper downstream genetic analysis by inhibiting enzymatic reactions in polymerase chain reaction (PCR; Weishing, Nybom, Wolff, & Meyer, 1995). Therefore, we used QiaAmp DNA stool kit (Qiagen Inc.) for DNA extractions from fecal pellets following minor modifications in the manufacturer's protocol (e.g., overnight incubation at 56°C with ASL buffer).

We used 64 nuclear microsatellite loci which have previously been used in population genetic studies of other lagomorph species (e.g., the European rabbit *Oryctolagus cuniculus*, Mougel, Mounolou, & Monnerot, 1997) and for other pika species (e.g., the American pika, Peacock, Kirchoff, & Merideth, 2002, Castillo et al., 2014; the Plateau pika *Ochotona curzoniae,* Li, Geng, Yang, Zhang, & Hu, 2009; and the Collared pika Zgurski, Davis, & Hik, 2009). Of these loci, seven polymorphic loci were retained to genotype samples, as mentioned in Table 1. We used Qiagen multiplex PCR kit and followed published protocols (Alves et al., 2015; Castillo et al., 2014; Li et al., 2009; Mougel et al., 1997; Peacock et al., 2002; Zgurski et al., 2009; see Table 1). The forward sequences of each primer were labeled with fluorescent dyes (FAM, NED, VIC, PET). The markers were multiplexed after the PCR amplification stage, as annealing temperature varied across loci. Each sample for each locus was amplified a minimum of four times to control for allelic dropout. Amplified products were diluted and run on an ABI 3730 automated sequencer and analyzed in GENEIOUS version 9.0.4 ( https://www.geneious.com/).

### 2.3 Data analyses

#### 2.3.1 Estimation of genetic diversity

The presence and frequency of null alleles and polymorphism information content (PIC) that indicate microsatellite primer's informativeness, mean number of alleles per locus (*A*), observed heterozygosity (*H*_O_), and expected heterozygosity (*H*_E_) were determined using CERVUS version 2.0 (Marshall, Slate, Kruuk, & Pemberton, 1998). We used Kruskal–Wallis rank sum test to check whether the *H*_O_ and *H*_E_ varied significantly across loci and sampled locations. To avoid analyzing multiple genotypes from the same individual in our final genotypic dataset, we assigned all samples to unique individuals using probability of identity statistics (PID; Paetkau et al., 1998) in CERVUS version 2.0. Any sample, which showed similarity at five loci, was considered a duplicate sample and was removed from the final dataset (see Cullingham et al., 2016). All loci were tested for departures from Hardy–Weinberg equilibrium (HWE) using CERVUS version 2.0 and used Holm's Bonferroni sequential corrections to adjust *p* values in HWE estimations (Gaetano, 2013). We tested for linkage disequilibrium using GENEPOP version 4.6 (Raymond, 1995; Rousset, 2008). We estimated likelihood of allelic dropout rate and false allele rate within each population with 10,000 search steps (Johnson & Haydon, 2007), using PEDANT version 1.0.

We used ESTIMATES version 9.0 with 10,000 iterations (Colwell et al., 2012) and the nonparametric Chao2 estimator (mean ± *SD*; Colwell & Coddington, 1994) to estimate our success in sampling alleles in populations (with more than 13 samples). We constructed rarefaction curves plotting the cumulative number of alleles found with increasing sample size.

Genetic divergence across populations (*F*_ST_) (Holsinger & Weir, 2009) and the inbreeding coefficient (*F*_IS_) (Weir & Cockerham, 1984) per population were estimated using ARLEQUIN version 3.5 (Excoffier & Lischer, 2010).

#### 2.3.2 Spatial autocorrelation

We examined the relationship between genetic relatedness and geographic distance using Wang's estimator “*r*” (Wang, 2002) in SPAGeDi version 1.4 (Hardy & Vekemans, 2002). This estimator is preferable among all relatedness indices due to its low sensitivity to sampling error in allele frequency calculation and has low sampling variance due to change in number of loci or alleles (Blouin 2003; Robinson, Simmons, & Kennington, 2013, Wang, 2017). The program estimates pairwise relatedness among each pair of individual pikas and regresses against pairwise straight‐line distance between them. In this study, the relatedness coefficients (Wang's estimator) were calculated against a range of distance classes (0.1, 0.5, 1, 5, 25, 50, 75, 100, 125 and >125 km). These classes were chosen to incorporate comparisons in local scale (0.1 km) as well as total spatial distance (>125 km) and to ensure minimum 100 individual pairwise comparisons per distance class to keep sample size large enough for robust statistical analysis (Hardy & Vekemans, 2002). The standard error for each distance class was estimated using a jackknife procedure over loci (Hardy & Vekemans, 2002), and deviation of Wang's estimate (*r*) from 0 suggests that Royle's pika individuals within a specific distance class are significantly related (positive values = high relatedness, negative values = low relatedness) and not random.

#### 2.3.3 Genetic differentiation in pika populations

We used five different methods to assess the population structure and differentiation in Royle's pika. The first two methods used a clustering approach: STRUCTURE and the discriminant analysis of principal components (DAPC) and three methods were based on distances: *F*_ST_ estimations, the analysis of the molecular variance (AMOVA), and isolation by distance (IBD). We included one locus that showed HWE deviation as the deviation was not observed in all populations.

We first investigated the likelihood of each individual sample belonging to one of several clusters (*K*) based on allele frequencies, using Bayesian clustering methodology in STRUCTURE version 2.3.4 (Pritchard, Stephens, & Donnelly, 2000). We implemented the admixture ancestry model with correlated allele frequencies. The putative numbers of population clusters (*K*) were allowed to vary from one to 10 with 50,000 burn‐in iterations and 500,000 Markov chain Monte Carlo (MCMC) iterations (Ishtiaq, Prakash, Green, & Johnson, 2015; Singh et al., 2017). A total of 20 independent runs were performed for each K (1–10) to achieve consistency across the runs. We used Structure Harvester (Earl & VonHoldt, 2012) to calculate Delta *K* (Evanno, Regnaut, & Goudet, 2005) in order to infer the optimal number of clusters. We used the program CLUMPAK ( https://clumpak.tau.ac.il/) to visualize the STRUCTURE results (the optimum number of populations, as estimated by the log likelihood and Delta *K*). STRUCTURE results often fail to detect fine‐scale hierarchical population structure (Janes et al., 2017). Therefore, we used a complementary multivariate analysis—discriminant analysis of principal components (DAPC; Jombart, Devillard, & Balloux, 2010) to detect complex spatial as well as hierarchical genetic structure across populations (Evanno et al., 2005; Jombart et al., 2010; Vergara et al., 2015). DAPC do not require the populations to fulfill common assumptions of traditional genetic models such as maintaining HWE or linkage equilibrium (LE) between loci (Jombart, 2008; Jombart et al., 2010). Thus, this multivariate analysis is often used as a robust tool to validate the inferences of individual Bayesian clustering software such as STRUCTURE.

We performed DAPC with the R package “Adegenet” (Jombart, 2008). DAPC define clusters using clustering algorithms *K*‐means on transformed data with principal component analysis (PCA). The clustering identifies groups of populations with similar genotypes by specifying the actual number of clusters (*K = *5) as a priori information (Putman & Carbone, 2014; Zachos et al., 2016). We retained 32 principal components of PCA, which explained approximately 90% of the total variation of our dataset. We used *q* > 0.7 to assign individuals to each location.

We used AMOVA to determine hierarchical distribution of genetic variation in ARLEQUIN. To run this analysis, we a priori defined five groups according to sampling sites and talus sampled within these sites. The significance of AMOVA was tested using 1,000 replicate bootstrap of all data.

To determine spatial patterns driving genetic differentiation among locations, linearized pairwise *F*_ST_ among talus (*F*_ST_/(1−*F*_ST_) obtained for seven microsatellite loci were correlated against log‐transformed geographic distances between talus using a Mantel test with 1,000 permutations in ARLEQUIN.

#### 2.3.4 Demographic effects of environmental conditions and habitat (bottleneck)

The detection of evidence for any population bottleneck was primarily based on Wilcoxon's signed rank test and shift in allelic distribution (Luikart, Allendorf, Cornuet, & Sherwin, 1998). We selected locations (TUN, MAD, and NAN) with sample size >10 individuals. We used BOTTLENECK version 1.2.02 to assess the genetic signature of demographic contractions using the heterozygote excess test (Cornuet & Luikart, 1996; Piry, Luikart, & Cornuet, 1999). The heterozygote excess test determines whether sudden decline in population size has led to the loss of rare alleles which can cause an expected heterozygosity excess (Luikart, Allendorf, et al., 1998; Luikart, Sherwin, Steele, & Allendorf, 1998; Nei, Maruyama, & Chakraborty, 1975). Evidence of a recent bottleneck in a population could be indicated by an increase in the number of heterozygotes relative to the number of alleles, whereas heterozygote deficiency indicates population growth (Cornuet & Luikart, 1996). Six out of seven microsatellite primers used in the study were dinucleotide. Hence, a two‐phase mutational model (TPM) with 95% single step mutations (SMM) and 12% variance among mutational steps, and 1,000 simulations for each location were used, which is generally recommended practice for dinucleotide microsatellite repeat loci (Di Rienzo et al., 1994; Piry et al., 1999).

## 3 RESULTS

### 3.1 Genetic diversity

A total of 203 fecal samples were collected from five locations. Of these samples, 68.96% were successfully amplified and produced consistent results across all replicates (Supporting Information Table S1). The average missing data across all loci were 2.95%, ranging from 0.71% (SAT3) to 7.14% (Ocp6). The rarefaction curve for allelic richness for each microsatellite locus indicated that we were able to detect all possible alleles across sampled populations (Supporting Information Table S2). No significant linkage disequilibrium was detected for any locus, whereas deviation from HW equilibrium was detected only in one out of seven loci in RUD and HAK populations after Bonferroni correction (Table 1). We retained all loci for further analysis as no systematic deviation of HWE was observed across all sites.

The observed allelic dropout rate (0.00–0.09) and false allele rate (0.00–0.07) were not significant for any locus. Null alleles were not detected in any population. The mean number of alleles per locus across all populations was 8.42. The mean polymorphism information content (PIC) was high (0.72) and varied from 0.40 to 0.85 indicating that loci were informative in detecting high levels of allelic polymorphism among genotyped individuals. (Table 1). The average expected heterozygosity across seven loci was 0.68, whereas mean observed heterozygosity was 0.57 (Table 2) and varied significantly across loci (Kruskal–Wallis chi‐squared = 5.58, *df* = 1, *p* < 0.01) and locations (Kruskal–Wallis chi‐squared = 6.091, *df* = 1, *p* < 0.01). The mean inbreeding coefficient (*F*_IS_) was 0.13 and varied from 0.01 to 0.50 across populations (Table 2).

**Table 2:** Genetic diversity across Royle's pika in the western Himalaya

Location	*T* a	*n*	*N*	A	*H*_O_	*H*_E_	*F*_IS_
HAK	14	6		2.71	0.33(0.09)	0.65(0.09)	0.50
MAD	7	13		4	0.61(0.09)	0.70(0.14)	0.08
TUN	56	79		5.71	0.67(0.11)	0.74(0.15)	0.08
RUD	5	7		3.57	0.63(0.19)	0.64(0.10)	0.01
NAN	22	35		4.85	0.65(0.11)	0.69(0.09)	0.04
Royles’ pika (this study)		140	7	4.16	0.57	0.68	0.13
American pika^b^		168	10	16.8	0.47	0.58	0.20
Collared pika^c^		442	15	5.8	0.61	0.62	0.03

A: mean number of alleles; *F*_IS_: mean inbreeding coefficient; *H*_E_: mean expected heterozygosity; *H*_O_: mean observed heterozygosity; *N*: number of loci used; *n*: number of samples successfully genotyped; *T*: total number of talus sampled during this study.

Standard deviation in parenthesis; location codes are the same as Figure 1

a Details of talus surveyed mentioned in Table S1

b Henry et al., 2012.

c Zgurski & Hik, 2014.

### 3.2 Spatial autocorrelation

**Figure 3:**
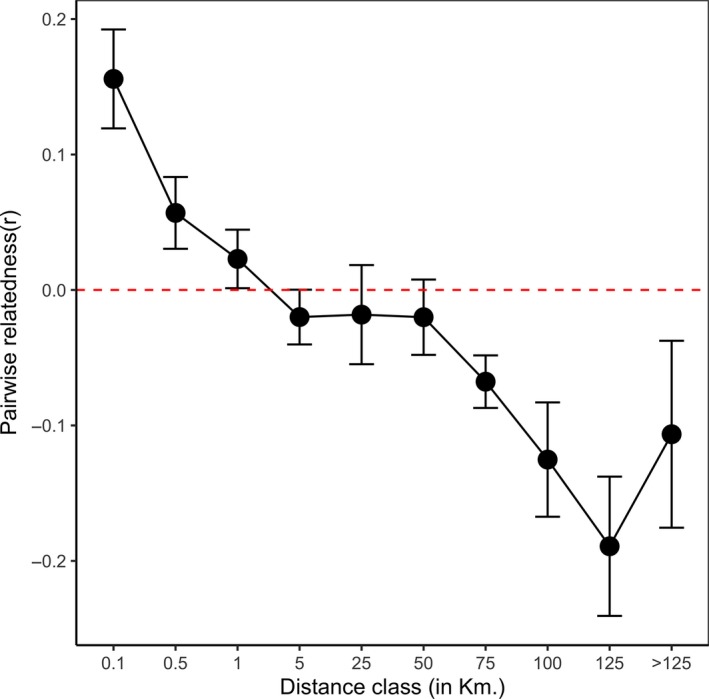
Average relatedness (Wang's *r*) between pairs of Royle's pika individuals in relation to their geographic proximity. The approximate standard errors obtained through jackknifing over loci are plotted as error bars

The pairwise relatedness analysis suggested local‐scale genetic structure in pika populations. The negative regression slope (*b* = −0.22 ± 0.005, *p* < 0.05) between Wang's estimator (*r*) and geographic distance suggested neighboring individuals within 1 km distance are more genetically related than any random pair of individuals (Figure 3).

### 3.3 Genetic differentiation in pika populations

**Figure 4:**
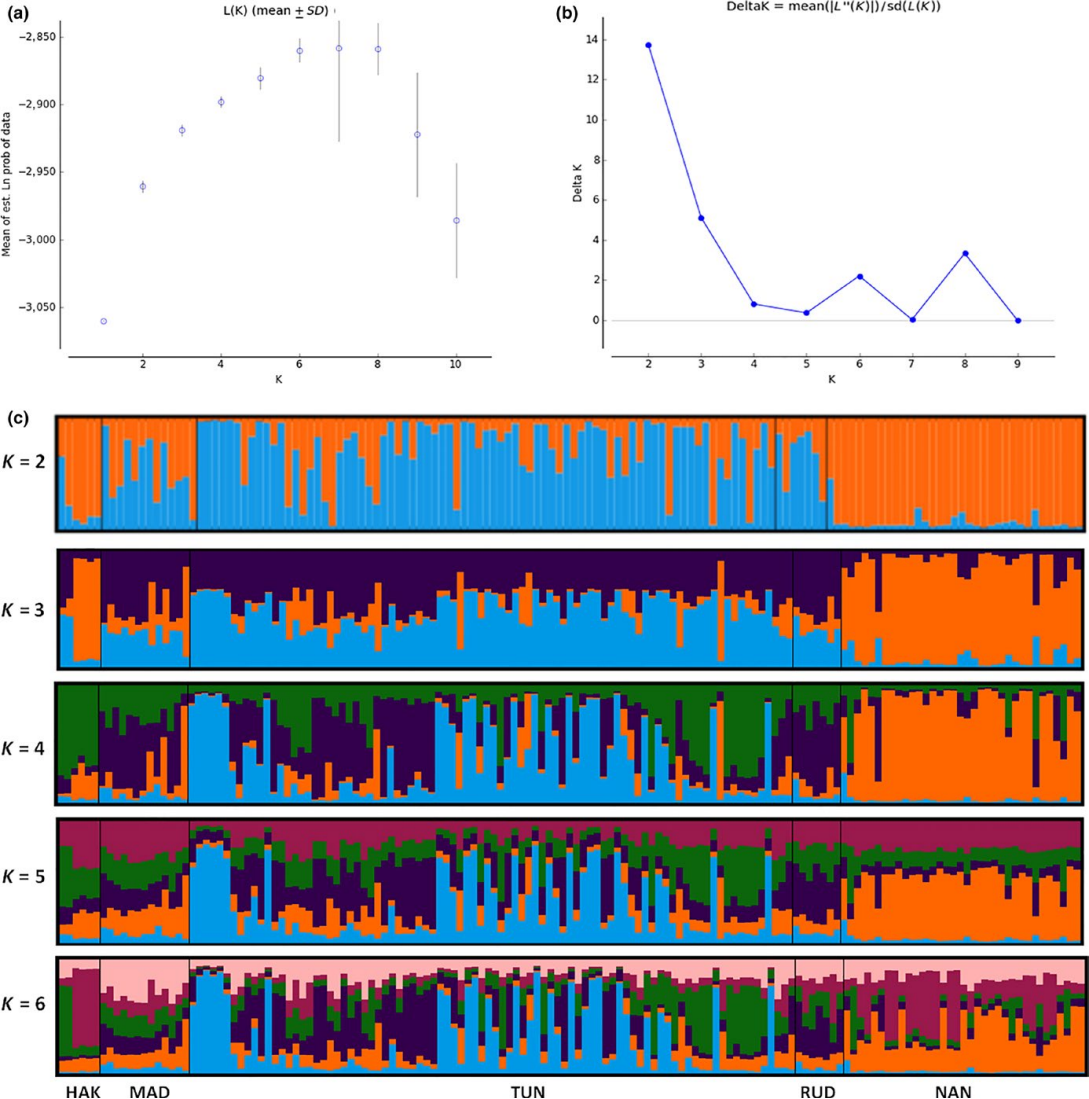
Nonspatial Bayesian individual‐based clustering STRUCTURE results from seven polymorphic microsatellite loci and admixture model. Plots showing the modal value of best *K* using maximum likelihood method (mean L (*K*) ± *SD*; a) and using Δ*K* method (b), the maximum Delta *K* = 13.70 was found at *K* = 2, followed by *K* = 3 with a Delta *K* = 5.12; visualization of the Bayesian clustering analysis implemented in STRUCTURE for *K* = 2–*K* = 6 (c) where each *K* is shown as a different color. Individuals are shown as single vertical bars, grouped by geographic location; location codes (below the plot) are the same as Figure 1. Data shown are aggregated data from 20 independent runs

The STRUCTURE results suggested a best fit of *K* = 2 clusters. The maximum Delta was found at *K* = 2, followed by *K* = 3 (Figure 4a,b). A large proportion of individuals (80%) were successfully assigned (*q* > 0.7) at *K* = 2 (Supporting Information Table S3), whereas at *K* = 3.5%, a large proportion of individuals were assigned (*q* > 0.7) to specific clusters. In *K* = 2, individuals in NAN (97%) and HAK (83.34%) were assigned in genetic cluster “A” and MAD (38.46%), TUN (69%), and RUD (57.14%) were assigned in cluster “B” (Figure 4c, Supporting Information Table S3). The DAPC suggested *K* = 3 as the most probable number of clusters which indicated genetic differentiation in HAK as a distinct cluster, whereas NAN, MAD, TUN, and RUD either partially or completely overlapped (Figure 2b).

The AMOVA suggested that a majority of the genetic variance was attributed to variation within individuals (84.45%), followed by 6.05% of the variation among locations, and 6.84% variation among talus within each location (Table 3). TUN and MAD were the only two locations with significantly low genetic differentiation (*F*_ST_ = 0.02, *p* < 0.01, Table 4), whereas other paired locations showed significantly high genetic differentiation (*F*_ST_ = 0.04−0.16, *p* < 0.01, Table 4).

**Table 3:** Analysis of molecular variance (AMOVA) for five sampled locations of Royle's pika

Source of variation	*df*	Sum of squares	Variance components	Percentage of variation	*p* Value
Among locations	4	33.22	0.13	6.05	<0.001
Among talus within locations	65	168.85	0.15	6.84	<0.001
Among individuals within talus	70	308.33	0.05	2.65	0.27
Within individuals	140	262.5	1.87	84.45	<0.001

*df*: degrees of freedom.

Significance tests were based on 1,000 permutations.

**Table 4:** Matrix of pairwise *F*_ST_ estimates (below diagonal) and their significance level (*P* value below 0.01*, 0.001**; above diagonal) across sampled locations of Royle’s pika in the western Himalaya

Location	HAK	MAD	TUN	RUD	NAN
HAK		**	**	**	**
MAD	0.13		**	*	**
TUN	0.13	0.02		**	**
RUD	0.19	0.04	0.04		**
NAN	0.12	0.04	0.06	0.07	

Note

Population codes are the same as Figure 1a.

The Mantel test showed significant evidence for a decrease in genetic differentiation across sampled individuals with an increase in geographic distance (*r* = 0.033, *p* < 0.001; Figure 2c).

### 3.4 Demographic change (bottleneck) in pika populations

We found significant (*p* < 0.05) heterozygosity excess across three locations suggesting that demographic change leads to a recent bottleneck (Table 5).

**Table 5:** Wilcoxon sign‐rank test and change in allelic distribution to detect heterozygosity excess due to significant reduction in Royle's pika population in the western Himalaya

Location	Wilcoxon's signed rank test probability		Allele frequency distribution
	TPM	SSM	
MAD	*p* = 0.003	*p* = 0.003	Shifted mode
TUN	*p* = 0.003	*p* = 0.003	Shifted mode
NAN	*p* = 0.03	*p* = 0.07	Shifted mode

Note

IAM: infinite alleles model; *p* = probability of significant heterozygosity excess; SMM: stepwise mutation model.

Location codes are the same as Figure 1a.

## 4 DISCUSSION

In this first genetic study on the Royle pika in south Asia, we used a noninvasive sampling approach where the quality of samples often relies on environmental conditions (Lucchini et al., 2002; Murphy, Kendall, Robinson, & Waits, 2007), sample preservation method (Nsubuga et al., 2004), and diet of model species (Panasci et al., 2011). Royle's pika occurs in open rocky talus habitat where fecal pellets were often exposed to high temperature fluctuation, high UV radiation, and extreme weather conditions, all of which could potentially degrade the DNA quality. Despite these constraints, we were able to successfully genotype large number (68.96%) of samples which showed high PIC value, low allelic dropout, and false allele rates to establish accuracy and reliability in our dataset.

### 4.1 Genetic variation and population structure in Royle's pika

Overall, the Royle's pika populations exhibited similar levels of genetic diversity (0.57) to those observed in other studies on talus‐dwelling *Ochotona* species (see Table 2). However, our genetic diversity values are not directly comparable as we used relatively fewer genetic markers than other studies. Nonetheless, the observed patterns in our data reflect pika life‐history traits rather than data quality. Natural populations with small numbers of individuals often lose genetic variability due to genetic drift and inbreeding (Lacy, 1997). The Royle's pika has a small litter size (Bhattacharyya et al., 2014a) and occurs at low density (16.2 individuals per ha). While there is no information on the extent of philopatry, our results indicate the possibility of inbreeding and a philopatric mode of dispersal with low inbreeding coefficients (*F*_IS_) and high level of pairwise relatedness in populations within 1 km. For a mainland mammal with broad geographic distribution, American pika showed similar patterns with low estimates of heterozygosity, low population density (around 10 individuals per ha) with high philopatry that suggest life‐history characteristics result in high within‐site inbreeding coefficients (Glover, Smith, Ames, Joule, & Dubach, 1977; Henry et al., 2012; Moilanen, Smith, & Hanski, 1998; Peacock, 1997; Peacock & Smith, 1997a; Smith & Ivins, 1983). The Royle's pika populations showed high genetic variation within individuals. Distribution of genetic diversity within and among populations is often found to be influenced by migration of individuals (Berthier, Charbonnel, Galan, Chaval, & Cosson, 2006; Hartl & Clark, 1997). Hence, populations with restricted movement and gene flow usually exhibit high genetic differentiation (Peery et al., 2008; Slarkin, 1985). The pairwise *F*_ST_ estimates across geographically closer locations (e.g., MAD and TUN or TUN and RUD; 8–13 km) indicated comparatively low genetic differentiation, suggesting more gene flow in recent times. This is in contrast to the pattern observed in the two geographically distant populations (HAK and NAN: 42–160 km), which showed moderate to very high genetic differentiation—so suggesting low gene flow (pairwise *F*_ST_ = 0.04–0.19). Furthermore, isolated mountain valley (cradle shaped) and absence of potential talus habitat near HAK location, which possibly reduces the gene flow from other pika populations, lead to high inbreeding coefficient. The genetic similarities within Royle's pika populations could be attributed to the following factors: (i) patchy distribution of talus habitat with suitable crevices (Bhattacharyya et al., 2015); (ii) preference toward endemic food plants with limited distribution range (Bhattacharyya et al., 2018); (iii) limited distribution of rock cover to protect from heat stress and predation risk (Bhattacharyya et al., 2014b); (iv) high interannual variation in the snow melting pattern, resulting in low survival and pika abundance (Bhattacharyya et al., 2014a).

Across two cluster approaches, DAPC results were in congruence with *F*_ST_ estimates due to limitation of STRUCTURE for detecting fine‐scale genetic structure (Janes et al, 2017). Landscape features and topography (e.g., elevation, aspect), habitat availability, and temperature gradient are factors which influence pika dispersal and connectivity between metapopulations over 10 km apart (Castillo et al., 2014, 2016 ; Henry et al., 2012; Robson, Lamb, & Russello, 2015; Smith, 1974). Royle's pika talus habitat is fragmented and cannot traverse physical barriers like wide valleys and river beds (e.g., Bhagirathi and Mandakini rivers form a barrier for HAK from other locations), which act as semipermeable barriers for dispersal and restrict distribution.

### 4.2 Effects of habitat and snow patterns on demography (population bottleneck)

We found evidence of a recent bottleneck, which is possibly a result of significant recent changes in habitat structure and rising winter temperature. The distribution and depth of snow in mountain regions are often influenced by wind and topography (e.g., aspect, slope), and microclimate, causing significant variation in snow cover, even across small geographical distances (Deems, Birkeland, & Hansen, 2002; Gurung et al., 2017). Royle's pika does not hibernate during winter and snow cover acts as a thermal insulator against extreme cold and fluctuating temperatures (Bhattacharyya et al., 2014a). While warm winters with thin snow cover can cause severe mortality in adult pikas, thin snow cover during the spring reduces reproductive fitness and thereby delays breeding dates (Bhattacharyya et al., 2014a; Morrison & Hik, 2007). In Royle's pika, talus size (area) and connectivity between talus are known to influence the habitat occupancy, whereas predation risk significantly impacts foraging ecology (Bhattacharyya et al., 2013, 2018, 2015 ) and potentially also access to nutritive plants, thereby affecting individual fitness. Furthermore, the decreased precipitation (3–4 mm per year from 1982 to 2006; Shrestha et al., 2012) during spring affects the growth rates of nutritive C_3_ plants in summer, which form the main diet of young pika (Bhattacharyya et al., 2018). Climate‐induced habitat loss and range contractions might be the main drivers for the apparent population bottleneck in pika. Contemporary climate change is expected to significantly affect the distribution and population dynamics in this talus‐dwelling species.

## 5 CONCLUSIONS

This is the first genetic study to establish the population structure of an important lagomorph species in the western Himalaya. Using clustering approaches, we found evidence for well‐defined population structure. We detected moderate levels of inbreeding and evidence for a recent population bottleneck and genetic isolation by geographic distance. We found little evidence of gene flow was observed between individuals >1 km apart. Therefore, being a climate‐sensitive small mammal which lives in isolated patches of mountain habitat and having limited dispersal ability, Royle's pika may depend on local adaptation in order to survive changing environmental conditions in the future. Our study highlights the need for an in‐depth study of this high‐altitude‐restricted model species and presents new evidence that it is imminently threatened by climate change.

## ETHICAL APPROVAL

The field experiments comply with the current laws of India where the study was performed. We thank Uttarakhand Forest Department for ethical approval and permission for collection of fecal samples.

## CONFLICT OF INTEREST

None declared.

## AUTHOR CONTRIBUTION

FI and SB designed the experiment. SB conducted the field and laboratory experiments. SB and FI analyzed the data and wrote the manuscript. Both authors approved the final version of the manuscript.

## DATA ACCESSIBILITY

The GPS locations of all sample collection points have already been added in Supporting Information. The microsatellite genotype data(allele frequency table) generated during the study has been submitted in the Dryad database (Provisional DOI: https://doi.org/10.5061/dryad.83jm680).
