# Supplementary material for: Noninvasive sampling reveals population genetic structure in the Royle’s pika, Ochotona roylei, in the western Himalaya
Source: Ecol Evol. 2018 Dec 26;9(1):180–91. doi: 10.1002/ece3.4707 (PMC6342111; doi:10.1002/ece3.4707)
Supplement: Supplementary file 1 [file ECE3-9-180-s001.docx]

**Supplementary material:**

**Noninvasive sampling reveals population genetic structure in the Royle’s pika *Ochotona roylei* in the western Himalaya**

Sabuj Bhattacharyya^1*^, Farah Ishtiaq^1^

^1^Centre for Ecological Sciences, Indian Institute of Science, Bangalore, Karnataka 560012, India

*Corresponding author: Email: bhattacharyyasabuj@gmail.com

Table S1: Summary of geographical location of faecal pellet collection, unique talus ID of samples successfully genotyped in this study.

| **Name of the Protected Area** | **Location** | **Talus no.** | **sample no.** | **Longitude** | **Latitude** |
| --- | --- | --- | --- | --- | --- |
| Govind Wildlife Sanctuary | HAK | H1 | 1 | 78.352629 | 31.117792 |
| Govind Wildlife Sanctuary | HAK | H1 | 2 | 78.36038439 | 31.119764 |
| Govind Wildlife Sanctuary | HAK | H2 | 3 | 78.42846941 | 31.15167925 |
| Govind Wildlife Sanctuary | HAK | H2 | 4 | 78.42846941 | 31.15167925 |
| Govind Wildlife Sanctuary | HAK | H3 | 5 | 78.42404088 | 31.14947128 |
| Govind Wildlife Sanctuary | HAK | H4 | 6 | 78.42307108 | 31.14849588 |
| Kedarnath Wildlife Sanctuary | MAD | M1 | 7 | 79.22061516 | 30.6354015 |
| Kedarnath Wildlife Sanctuary | MAD | M2 | 8 | 79.22046352 | 30.63570616 |
| Kedarnath Wildlife Sanctuary | MAD | M2 | 9 | 79.22046352 | 30.63570616 |
| Kedarnath Wildlife Sanctuary | MAD | M3 | 10 | 79.21998733 | 30.6349328 |
| Kedarnath Wildlife Sanctuary | MAD | M4 | 11 | 79.21945276 | 30.63563819 |
| Kedarnath Wildlife Sanctuary | MAD | M4 | 12 | 79.21945276 | 30.63563819 |
| Kedarnath Wildlife Sanctuary | MAD | M5 | 13 | 79.21914648 | 30.63584149 |
| Kedarnath Wildlife Sanctuary | MAD | M5 | 14 | 79.21914648 | 30.63584149 |
| Kedarnath Wildlife Sanctuary | MAD | M6 | 15 | 79.22041673 | 30.63712191 |
| Kedarnath Wildlife Sanctuary | MAD | M7 | 16 | 79.22057236 | 30.63716915 |
| Kedarnath Wildlife Sanctuary | MAD | M7 | 17 | 79.22057236 | 30.63716915 |
| Kedarnath Wildlife Sanctuary | MAD | M7 | 18 | 79.22057236 | 30.63716915 |
| Kedarnath Wildlife Sanctuary | MAD | M7 | 19 | 79.22057236 | 30.63716915 |
| Kedarnath Wildlife Sanctuary | TUN | T1 | 20 | 79.21809006 | 30.49341933 |
| Kedarnath Wildlife Sanctuary | TUN | T1 | 21 | 79.21809006 | 30.49341933 |
| Kedarnath Wildlife Sanctuary | TUN | T1 | 22 | 79.21809006 | 30.49341933 |
| Kedarnath Wildlife Sanctuary | TUN | T2 | 23 | 79.21790423 | 30.49332657 |
| Kedarnath Wildlife Sanctuary | TUN | T2 | 24 | 79.21790423 | 30.49332657 |
| Kedarnath Wildlife Sanctuary | TUN | T2 | 25 | 79.2176092 | 30.49236623 |
| Kedarnath Wildlife Sanctuary | TUN | T2 | 26 | 79.21768671 | 30.49211468 |
| Kedarnath Wildlife Sanctuary | TUN | T3 | 27 | 79.21795745 | 30.49155001 |
| Kedarnath Wildlife Sanctuary | TUN | T3 | 28 | 79.21795745 | 30.49155001 |
| Kedarnath Wildlife Sanctuary | TUN | T3 | 29 | 79.21805299 | 30.49145207 |
| Kedarnath Wildlife Sanctuary | TUN | T3 | 30 | 79.21798804 | 30.49158651 |
| Kedarnath Wildlife Sanctuary | TUN | T3 | 31 | 79.21795745 | 30.49155001 |
| Kedarnath Wildlife Sanctuary | TUN | T4 | 32 | 79.21926698 | 30.4905755 |
| Kedarnath Wildlife Sanctuary | TUN | T5 | 33 | 79.21687008 | 30.49062393 |
| Kedarnath Wildlife Sanctuary | TUN | T6 | 34 | 79.21745419 | 30.48944105 |
| Kedarnath Wildlife Sanctuary | TUN | T7 | 35 | 79.22082189 | 30.48641964 |
| Kedarnath Wildlife Sanctuary | TUN | T7 | 36 | 79.22136131 | 30.48654428 |
| Kedarnath Wildlife Sanctuary | TUN | T7 | 37 | 79.22144676 | 30.48642817 |
| Kedarnath Wildlife Sanctuary | TUN | T8 | 38 | 79.21457474 | 30.48910392 |
| Kedarnath Wildlife Sanctuary | TUN | T9 | 39 | 79.21098959 | 30.48862172 |
| Kedarnath Wildlife Sanctuary | TUN | T9 | 40 | 79.21098959 | 30.48862172 |
| Kedarnath Wildlife Sanctuary | TUN | T9 | 41 | 79.21098959 | 30.48862172 |
| Kedarnath Wildlife Sanctuary | TUN | T10 | 42 | 79.20934705 | 30.48843677 |
| Kedarnath Wildlife Sanctuary | TUN | T10 | 43 | 79.20934705 | 30.48843677 |
| Kedarnath Wildlife Sanctuary | TUN | T10 | 44 | 79.20926324 | 30.48846269 |
| Kedarnath Wildlife Sanctuary | TUN | T11 | 45 | 79.20868944 | 30.48794056 |
| Kedarnath Wildlife Sanctuary | TUN | T11 | 46 | 79.20821186 | 30.4878528 |
| Kedarnath Wildlife Sanctuary | TUN | T11 | 47 | 79.20821186 | 30.4878528 |
| Kedarnath Wildlife Sanctuary | TUN | T11 | 48 | 79.20821186 | 30.4878528 |
| Kedarnath Wildlife Sanctuary | TUN | T12 | 49 | 79.2085643 | 30.48851623 |
| Kedarnath Wildlife Sanctuary | TUN | T12 | 50 | 79.20842048 | 30.488406 |
| Kedarnath Wildlife Sanctuary | TUN | T12 | 51 | 79.20842048 | 30.488406 |
| Kedarnath Wildlife Sanctuary | TUN | T12 | 52 | 79.20842048 | 30.488406 |
| Kedarnath Wildlife Sanctuary | TUN | T13 | 53 | 79.2082917 | 30.48861173 |
| Kedarnath Wildlife Sanctuary | TUN | T13 | 54 | 79.20842048 | 30.488406 |
| Kedarnath Wildlife Sanctuary | TUN | T13 | 55 | 79.20833683 | 30.48842289 |
| Kedarnath Wildlife Sanctuary | TUN | T14 | 56 | 79.20863671 | 30.48911267 |
| Kedarnath Wildlife Sanctuary | TUN | T14 | 57 | 79.20861489 | 30.4891665 |
| Kedarnath Wildlife Sanctuary | TUN | T14 | 58 | 79.20904553 | 30.48954231 |
| Kedarnath Wildlife Sanctuary | TUN | T14 | 59 | 79.20904553 | 30.48954231 |
| Kedarnath Wildlife Sanctuary | TUN | T15 | 60 | 79.20876781 | 30.48991741 |
| Kedarnath Wildlife Sanctuary | TUN | T15 | 61 | 79.20856745 | 30.49004998 |
| Kedarnath Wildlife Sanctuary | TUN | T16 | 62 | 79.20754763 | 30.48942248 |
| Kedarnath Wildlife Sanctuary | TUN | T16 | 63 | 79.20718593 | 30.48926414 |
| Kedarnath Wildlife Sanctuary | TUN | T17 | 64 | 79.20722759 | 30.48926471 |
| Kedarnath Wildlife Sanctuary | TUN | T17 | 65 | 79.20712856 | 30.48955204 |
| Kedarnath Wildlife Sanctuary | TUN | T18 | 66 | 79.20687828 | 30.48956664 |
| Kedarnath Wildlife Sanctuary | TUN | T19 | 67 | 79.20689845 | 30.48960301 |
| Kedarnath Wildlife Sanctuary | TUN | T19 | 68 | 79.20677397 | 30.48957423 |
| Kedarnath Wildlife Sanctuary | TUN | T20 | 69 | 79.20609501 | 30.49024152 |
| Kedarnath Wildlife Sanctuary | TUN | T21 | 70 | 79.20603136 | 30.4903038 |
| Kedarnath Wildlife Sanctuary | TUN | T21 | 71 | 79.20603136 | 30.4903038 |
| Kedarnath Wildlife Sanctuary | TUN | T22 | 72 | 79.20585575 | 30.49249367 |
| Kedarnath Wildlife Sanctuary | TUN | T23 | 73 | 79.20578284 | 30.49249267 |
| Kedarnath Wildlife Sanctuary | TUN | T23 | 74 | 79.20811299 | 30.49495158 |
| Kedarnath Wildlife Sanctuary | TUN | T23 | 75 | 79.20822971 | 30.4948359 |
| Kedarnath Wildlife Sanctuary | TUN | T24 | 76 | 79.20822971 | 30.4948359 |
| Kedarnath Wildlife Sanctuary | TUN | T24 | 77 | 79.20375152 | 30.49021827 |
| Kedarnath Wildlife Sanctuary | TUN | T25 | 78 | 79.2016663 | 30.48691462 |
| Kedarnath Wildlife Sanctuary | TUN | T25 | 79 | 79.2016663 | 30.48691462 |
| Kedarnath Wildlife Sanctuary | TUN | T26 | 80 | 79.20064029 | 30.4883259 |
| Kedarnath Wildlife Sanctuary | TUN | T26 | 81 | 79.2006076 | 30.48783827 |
| Kedarnath Wildlife Sanctuary | TUN | T27 | 82 | 79.20088908 | 30.48839248 |
| Kedarnath Wildlife Sanctuary | TUN | T27 | 83 | 79.19978177 | 30.489126 |
| Kedarnath Wildlife Sanctuary | TUN | T28 | 84 | 79.19985201 | 30.48927132 |
| Kedarnath Wildlife Sanctuary | TUN | T28 | 85 | 79.19812881 | 30.49402904 |
| Kedarnath Wildlife Sanctuary | TUN | T29 | 86 | 79.19470348 | 30.48488772 |
| Kedarnath Wildlife Sanctuary | TUN | T29 | 87 | 79.19507655 | 30.48499213 |
| Kedarnath Wildlife Sanctuary | TUN | T29 | 88 | 79.194961 | 30.48504466 |
| Kedarnath Wildlife Sanctuary | TUN | T30 | 89 | 79.19498991 | 30.48517136 |
| Kedarnath Wildlife Sanctuary | TUN | T30 | 90 | 79.19736294 | 30.48132484 |
| Kedarnath Wildlife Sanctuary | TUN | T30 | 91 | 79.18902248 | 30.48228284 |
| Kedarnath Wildlife Sanctuary | TUN | T31 | 92 | 79.18902415 | 30.48219264 |
| Kedarnath Wildlife Sanctuary | TUN | T31 | 93 | 79.20329238 | 30.48119017 |
| Kedarnath Wildlife Sanctuary | TUN | T31 | 94 | 79.20329238 | 30.48119017 |
| Kedarnath Wildlife Sanctuary | TUN | T32 | 95 | 79.20286939 | 30.48096782 |
| Kedarnath Wildlife Sanctuary | TUN | T32 | 96 | 79.20406037 | 30.48077673 |
| Kedarnath Wildlife Sanctuary | TUN | T33 | 97 | 79.219372 | 30.470485 |
| Kedarnath Wildlife Sanctuary | TUN | T34 | 98 | 79.227829 | 30.460162 |
| Kedarnath Wildlife Sanctuary | RUD | R1 | 99 | 79.31213371 | 30.49335387 |
| Kedarnath Wildlife Sanctuary | RUD | R1 | 100 | 79.31213371 | 30.49335387 |
| Kedarnath Wildlife Sanctuary | RUD | R2 | 101 | 79.31122419 | 30.49172715 |
| Kedarnath Wildlife Sanctuary | RUD | R3 | 102 | 79.31853107 | 30.51934779 |
| Kedarnath Wildlife Sanctuary | RUD | R4 | 103 | 79.33434009 | 30.48080112 |
| Kedarnath Wildlife Sanctuary | RUD | R5 | 104 | 79.33434009 | 30.48080112 |
| Kedarnath Wildlife Sanctuary | RUD | R5 | 105 | 79.33434009 | 30.48080112 |
| NandaDevi Biosphere Reserve | NAN | N1 | 106 | 79.66660626 | 30.20604866 |
| NandaDevi Biosphere Reserve | NAN | N1 | 107 | 79.66667897 | 30.20604939 |
| NandaDevi Biosphere Reserve | NAN | N2 | 108 | 79.66670903 | 30.20613091 |
| NandaDevi Biosphere Reserve | NAN | N3 | 109 | 79.66722291 | 30.20654217 |
| NandaDevi Biosphere Reserve | NAN | N3 | 110 | 79.66722291 | 30.20654217 |
| NandaDevi Biosphere Reserve | NAN | N3 | 111 | 79.66722291 | 30.20654217 |
| NandaDevi Biosphere Reserve | NAN | N4 | 112 | 79.66742944 | 30.2066345 |
| NandaDevi Biosphere Reserve | NAN | N5 | 113 | 79.66785713 | 30.2065035 |
| NandaDevi Biosphere Reserve | NAN | N6 | 114 | 79.66757352 | 30.20673522 |
| NandaDevi Biosphere Reserve | NAN | N6 | 115 | 79.66757352 | 30.20673522 |
| NandaDevi Biosphere Reserve | NAN | N7 | 116 | 79.66776529 | 30.20560928 |
| NandaDevi Biosphere Reserve | NAN | N7 | 117 | 79.66776529 | 30.20560928 |
| NandaDevi Biosphere Reserve | NAN | N7 | 118 | 79.66776529 | 30.20560928 |
| NandaDevi Biosphere Reserve | NAN | N8 | 119 | 79.66716188 | 30.20567533 |
| NandaDevi Biosphere Reserve | NAN | N8 | 120 | 79.66716188 | 30.20567533 |
| NandaDevi Biosphere Reserve | NAN | N9 | 121 | 79.67165166 | 30.20707437 |
| NandaDevi Biosphere Reserve | NAN | N9 | 122 | 79.67165166 | 30.20707437 |
| NandaDevi Biosphere Reserve | NAN | N9 | 123 | 79.67165166 | 30.20707437 |
| NandaDevi Biosphere Reserve | NAN | N10 | 124 | 79.67275558 | 30.21770576 |
| NandaDevi Biosphere Reserve | NAN | N11 | 125 | 79.67285506 | 30.20874682 |
| NandaDevi Biosphere Reserve | NAN | N12 | 126 | 79.67308768 | 30.20921837 |
| NandaDevi Biosphere Reserve | NAN | N12 | 127 | 79.67252833 | 30.20909541 |
| NandaDevi Biosphere Reserve | NAN | N13 | 128 | 79.67239257 | 30.20914817 |
| NandaDevi Biosphere Reserve | NAN | N14 | 129 | 79.67265707 | 30.20956592 |
| NandaDevi Biosphere Reserve | NAN | N15 | 130 | 79.69196955 | 30.23550306 |
| NandaDevi Biosphere Reserve | NAN | N15 | 131 | 79.69196955 | 30.23550306 |
| NandaDevi Biosphere Reserve | NAN | N15 | 132 | 79.69196955 | 30.23550306 |
| NandaDevi Biosphere Reserve | NAN | N16 | 133 | 79.69357255 | 30.23922756 |
| NandaDevi Biosphere Reserve | NAN | N17 | 134 | 79.69251378 | 30.23756576 |
| NandaDevi Biosphere Reserve | NAN | N18 | 135 | 79.68359116 | 30.26143271 |
| NandaDevi Biosphere Reserve | NAN | N18 | 136 | 79.68359116 | 30.26143271 |
| NandaDevi Biosphere Reserve | NAN | N19 | 137 | 79.67619918 | 30.20804976 |
| NandaDevi Biosphere Reserve | NAN | N19 | 138 | 79.67341146 | 30.20599137 |
| NandaDevi Biosphere Reserve | NAN | N20 | 139 | 79.67338127 | 30.20591888 |
| NandaDevi Biosphere Reserve | NAN | N20 | 140 | 79.67338127 | 30.20591888 |

Table S2. Estimation of the non-parametric Chao2 (mean± standard deviation) for allele richness captured by seven microsatellite loci in three Royle’s pika locations [TUN (n=79), MAD (n=13), NAN (n=35); HAK, RUD were not considered due to low sample size] in the Western Himalaya. (N=Total allele detected during current study; location codes are the same as Fig. 1A)

| Loci | MAD | | TUN | | NAN | |
| --- | --- | --- | --- | --- | --- | --- |
|  | N | Chao2 (mean± SD) | N | Chao2 (mean± SD) | N | Chao2 (mean± SD) |
| OCP16 | 3 | 1.93±0.16 | 5 | 1.96±0.02 | 5 | 1.84±0.24 |
| OCP6 | 2 | 2.52±0.31 | 2 | 4.77±0.36 | 3 | 4.25±0.57 |
| P7 | 5 | 4.61±0.76 | 8 | 7.53±0.62 | 8 | 7.31±1.16 |
| R106 | 7 | 6.63±1.62 | 9 | 8.56±0.89 | 6 | 5.65±0.67 |
| STR14 | 4 | 3.72±0.54 | 10 | 9.01±1.54 | 6 | 5.33±0.70 |
| SAT4 | 4 | 3.77±0.57 | 10 | 9.06±1.35 | 3 | 2.88±0.19 |
| STR31 | 3 | 2.82±0.31 | 9 | 8.11±1.27 | 5 | 4.28±0.69 |

Table S3. Assignment of individuals (with Q>0.7) to unique genetic clusters based on optimum number of cluster *K*=2 suggested by STRUCTURE (location codes are the same as Fig. 1A)

| **Population** | **Individual assignment  to genetic clusters(K=2)** | |
| --- | --- | --- |
|  | **Cluster A** | **Cluster B** |
| HAK | 0.3416 | 0.6584 |
| HAK | 0.7834 | 0.2167 |
| HAK | 0.9116 | 0.0885 |
| HAK | 0.9462 | 0.0538 |
| HAK | 0.8798 | 0.1202 |
| HAK | 0.8838 | 0.1162 |
| MAD | 0.0647 | 0.9353 |
| MAD | 0.7133 | 0.2866 |
| MAD | 0.6085 | 0.3915 |
| MAD | 0.1906 | 0.8094 |
| MAD | 0.5594 | 0.4407 |
| MAD | 0.2496 | 0.7504 |
| MAD | 0.184 | 0.816 |
| MAD | 0.7544 | 0.2457 |
| MAD | 0.2175 | 0.7826 |
| MAD | 0.627 | 0.373 |
| MAD | 0.5598 | 0.4402 |
| MAD | 0.5242 | 0.4758 |
| MAD | 0.9141 | 0.0859 |
| TUN | 0.0292 | 0.9708 |
| TUN | 0.0243 | 0.9757 |
| TUN | 0.0165 | 0.9835 |
| TUN | 0.0293 | 0.9707 |
| TUN | 0.017 | 0.9831 |
| TUN | 0.0383 | 0.9617 |
| TUN | 0.5999 | 0.4001 |
| TUN | 0.1646 | 0.8354 |
| TUN | 0.0262 | 0.9738 |
| TUN | 0.0402 | 0.9598 |
| TUN | 0.2361 | 0.7638 |
| TUN | 0.0399 | 0.9602 |
| TUN | 0.795 | 0.2049 |
| TUN | 0.2812 | 0.7188 |
| TUN | 0.8672 | 0.1328 |
| TUN | 0.4559 | 0.5441 |
| TUN | 0.1694 | 0.8306 |
| TUN | 0.7473 | 0.2527 |
| TUN | 0.9677 | 0.0323 |
| TUN | 0.3021 | 0.6979 |
| TUN | 0.1881 | 0.8119 |
| TUN | 0.1257 | 0.8742 |
| TUN | 0.352 | 0.6481 |
| TUN | 0.6086 | 0.3914 |
| TUN | 0.7718 | 0.2283 |
| TUN | 0.1537 | 0.8463 |
| TUN | 0.0305 | 0.9696 |
| TUN | 0.3248 | 0.6752 |
| TUN | 0.0241 | 0.9759 |
| TUN | 0.3671 | 0.6329 |
| TUN | 0.0297 | 0.9703 |
| TUN | 0.0598 | 0.9402 |
| TUN | 0.1367 | 0.8633 |
| TUN | 0.055 | 0.945 |
| TUN | 0.0483 | 0.9517 |
| TUN | 0.821 | 0.179 |
| TUN | 0.0285 | 0.9715 |
| TUN | 0.0411 | 0.9589 |
| TUN | 0.2706 | 0.7294 |
| TUN | 0.1089 | 0.8911 |
| TUN | 0.1011 | 0.899 |
| TUN | 0.2083 | 0.7917 |
| TUN | 0.1262 | 0.8738 |
| TUN | 0.0472 | 0.9528 |
| TUN | 0.423 | 0.577 |
| TUN | 0.4787 | 0.5213 |
| TUN | 0.0528 | 0.9473 |
| TUN | 0.0549 | 0.9452 |
| TUN | 0.2735 | 0.7265 |
| TUN | 0.4911 | 0.5089 |
| TUN | 0.0565 | 0.9435 |
| TUN | 0.359 | 0.641 |
| TUN | 0.1111 | 0.889 |
| TUN | 0.0275 | 0.9725 |
| TUN | 0.0744 | 0.9255 |
| TUN | 0.1036 | 0.8964 |
| TUN | 0.5323 | 0.4677 |
| TUN | 0.1683 | 0.8318 |
| TUN | 0.5133 | 0.4867 |
| TUN | 0.0313 | 0.9688 |
| TUN | 0.3899 | 0.6101 |
| TUN | 0.0169 | 0.9831 |
| TUN | 0.0526 | 0.9474 |
| TUN | 0.0429 | 0.9572 |
| TUN | 0.8668 | 0.1333 |
| TUN | 0.0749 | 0.9251 |
| TUN | 0.0785 | 0.9214 |
| TUN | 0.0795 | 0.9205 |
| TUN | 0.245 | 0.7549 |
| TUN | 0.0366 | 0.9634 |
| TUN | 0.9584 | 0.0416 |
| TUN | 0.3598 | 0.6402 |
| TUN | 0.5451 | 0.4549 |
| TUN | 0.0435 | 0.9565 |
| TUN | 0.1818 | 0.8182 |
| TUN | 0.9381 | 0.062 |
| TUN | 0.2752 | 0.7248 |
| TUN | 0.0438 | 0.9562 |
| TUN | 0.44 | 0.56 |
| RUD | 0.1381 | 0.8619 |
| RUD | 0.6595 | 0.3405 |
| RUD | 0.2202 | 0.7798 |
| RUD | 0.1608 | 0.8392 |
| RUD | 0.1185 | 0.8815 |
| RUD | 0.3114 | 0.6886 |
| RUD | 0.7426 | 0.2574 |
| NAN | 0.5394 | 0.4606 |
| NAN | 0.9501 | 0.0499 |
| NAN | 0.9456 | 0.0544 |
| NAN | 0.9746 | 0.0254 |
| NAN | 0.969 | 0.031 |
| NAN | 0.9768 | 0.0232 |
| NAN | 0.9621 | 0.0379 |
| NAN | 0.9609 | 0.0392 |
| NAN | 0.9607 | 0.0393 |
| NAN | 0.9597 | 0.0403 |
| NAN | 0.9566 | 0.0434 |
| NAN | 0.9632 | 0.0368 |
| NAN | 0.8038 | 0.1962 |
| NAN | 0.9235 | 0.0765 |
| NAN | 0.9736 | 0.0265 |
| NAN | 0.9627 | 0.0373 |
| NAN | 0.9551 | 0.0449 |
| NAN | 0.8804 | 0.1196 |
| NAN | 0.8416 | 0.1585 |
| NAN | 0.9435 | 0.0565 |
| NAN | 0.9633 | 0.0368 |
| NAN | 0.9813 | 0.0187 |
| NAN | 0.9616 | 0.0384 |
| NAN | 0.9435 | 0.0565 |
| NAN | 0.9353 | 0.0647 |
| NAN | 0.9184 | 0.0816 |
| NAN | 0.9502 | 0.0498 |
| NAN | 0.9782 | 0.0219 |
| NAN | 0.8181 | 0.1819 |
| NAN | 0.9687 | 0.0313 |
| NAN | 0.9677 | 0.0323 |
| NAN | 0.9595 | 0.0405 |
| NAN | 0.9516 | 0.0484 |
| NAN | 0.9809 | 0.0192 |
| NAN | 0.9738 | 0.0263 |
|  | Total % of  individual assignment (Q>0.7) | |
|  | Cluster A | Cluster B |
| HAK | 83.34 | 16.66 |
| MAD | 15.38 | 38.46 |
| TUN | 7 | 69 |
| RUD | 14.28 | 57.14 |
| NAN | 97 | 0 |
